# Transcript profiling of structural genes involved in cyanidin-based anthocyanin biosynthesis between purple and non-purple carrot (*Daucus carota* L.) cultivars reveals distinct patterns

**DOI:** 10.1186/s12870-014-0262-y

**Published:** 2014-10-01

**Authors:** Zhi-Sheng Xu, Ying Huang, Feng Wang, Xiong Song, Guang-Long Wang, Ai-Sheng Xiong

**Affiliations:** State Key Laboratory of Crop Genetics and Germplasm Enhancement, College of Horticulture, Nanjing Agricultural University, Nanjing, 210095 China

**Keywords:** Anthocyanin pathway, Root development, Purple carrot, Cyanidin, *Daucus carota* L, Gene expression

## Abstract

**Background:**

Carrots (*Daucus carota* L.) are among the 10 most economically important vegetable crops grown worldwide. Purple carrot cultivars accumulate rich cyanidin-based anthocyanins in a light-independent manner in their taproots whereas other carrot color types do not. Anthocyanins are important secondary metabolites in plants, protecting them from damage caused by strong light, heavy metals, and pathogens. Furthermore, they are important nutrients for human health. Molecular mechanisms underlying anthocyanin accumulation in purple carrot cultivars and loss of anthocyanin production in non-purple carrot cultivars remain unknown.

**Results:**

The taproots of the three purple carrot cultivars were rich in anthocyanin, and levels increased during development. Conversely, the six non-purple carrot cultivars failed to accumulate anthocyanins in the underground part of taproots. Six novel structural genes, *CA4H1*, *CA4H2*, *4CL1*, *4CL2*, *CHI1*, and *F3′H1*, were isolated from purple carrots. The expression profiles of these genes, together with other structural genes known to be involved in anthocyanin biosynthesis, were analyzed in three purple and six non-purple carrot cultivars at the 60-day-old stage. *PAL3/PAL4*, *CA4H1*, and *4CL1* expression levels were higher in purple than in non-purple carrot cultivars. Expression of *CHS1*, *CHI1*, *F3H1*, *F3′H1*, *DFR1*, and *LDOX1/LDOX2* was highly correlated with the presence of anthocyanin as these genes were highly expressed in purple carrot taproots but not or scarcely expressed in non-purple carrot taproots.

**Conclusions:**

This study isolated six novel structural genes involved in anthocyanin biosynthesis in carrots. Among the 13 analyzed structural genes, *PAL3/PAL4*, *CA4H1*, *4CL1*, *CHS1*, *CHI1*, *F3H1*, *F3′H1*, *DFR1*, and *LDOX1/LDOX2* may participate in anthocyanin biosynthesis in the taproots of purple carrot cultivars. *CHS1*, *CHI1*, *F3H1*, *F3′H1*, *DFR1*, and *LDOX1/LDOX2* may lead to loss of light-independent anthocyanin production in orange and yellow carrots. These results suggest that numerous structural genes are involved in anthocyanin production in the taproots of purple carrot cultivars and in the loss of anthocyanin production in non-purple carrots. Unexpressed or scarcely expressed genes in the taproots of non-purple carrot cultivars may be caused by the inactivation of regulator genes. Our results provide new insights into anthocyanin biosynthesis at the molecular level in carrots and for other root vegetables.

**Electronic supplementary material:**

The online version of this article (doi:10.1186/s12870-014-0262-y) contains supplementary material, which is available to authorized users.

## Background

Anthocyanins are widely distributed water-soluble pigments belonging to the flavanoid group of phytochemicals. Over 635 types of anthocyanins have been identified [1]. These mainly possess six common aglycones (cyanidin, pelargonidin, delphinidin, peonidin, petunidin, and malvidin) and various types of glycosylated and acylated compounds [2]. Anthocyanins protect plants from strong light, heavy metals, and pathogens, and play an important role in flowers [3,4]. As they have low toxicity and vary in color they are often used as healthier alternatives to synthetic colorants [5]. Previous studies have confirmed that anthocyanins provide antioxidants for human health that protect against a broad range of diseases, including a high blood cholesterol level, cardiovascular disease, and ultraviolet radiation damage [2,6-8].

Carrots (*Daucus carota* L.) are among the 10 most economically important vegetable crops grown worldwide [9]. Carrot cultivars appear in five taproot color types: purple, orange, yellow, red, and white. Although orange carrot cultivars (*D. carota* ssp. *sativus* var.*sativus*) account for the majority of production, purple carrots (*D. carota* ssp. *sativus* var.*atrorubens* Alef.) are enjoying increased popularity, largely because they contain high amounts of anthocyanin in their flesh taproots. Purple carrot cultivars have existed for over 3000 years, and are much older than orange carrot cultivars [10]. Anthocyanins from purple carrots are commonly used as natural food colorants in candies, ice cream, and beverages, this is because they remain stable when exposed to heat and light, and have increased pH values [11,12]. Purple carrots mainly contain cyanidin-based anthocyanins; some cultivars also contain trace amounts of peonidin- or pelargonidin-based anthocyanins in their taproots [13].

The anthocyanin biosynthesis pathway has been extensively studied in numerous plant species, including bilberry (*Vaccinium myrtillus* L.), grape (*Vitis vinifera* L.), apple (*Malus* × *domestica*), *Arabidopsis* (*Arabidopsis thaliana*), Mitchell petunia [*Petunia axillaris* × (*Petunia axillaris* × *Petunia hybrida* cv. ‘Rose of Heaven’)], and sweet potato (*Ipomoea batatas* L. Lam.) [14-20]. Two classes of genes participate in the anthocyanin biosynthesis pathway: structural genes and regulatory genes. Structural genes encode enzymes that directly catalyze reaction steps leading to the formation of anthocyanins; the transcription of these genes is controlled by regulatory genes, such as *MYB*, *bHLH*, and *WD40* genes [16,17,21]. Structural genes that participate in anthocyanin biosynthesis have been identified in mumerous plant species [14-18,20-23]. Some functional genes participating in this pathway have also been identified in carrots; these include the phenylalanine ammonia-lyase (PAL), chalcone synthase (CHS), flavanone 3-hydroxylase (F3H), dihydroflavonol 4-reductase (DFR), and leucoanthocyanidin dioxygenase (LDOX) genes [24]. We described the presence of the UDP-galactose: cyanidin 3-*O*-galactosyltransferase (UCGT) in the purple carrot cultivars in our previous study [25]. However, cinnamate 4-hydroxylase (CA4H), 4-coumaroyl-coenzyme A ligase (4CL), chalcone-flavonone isomerase (CHI), and flavonoid 3'-hydroxylase (F3′H) genes have not been identified in carrots.

To obtain insights into differences in anthocyanin biosynthesis between purple and non-purple carrot cultivars, we cloned six novel structural genes involved in anthocyanin biosyntheses from taproot-derived cDNA. The expression patterns of 13 structural genes in the taproots of three purple and six non-purple carrot cultivars were analyzed at the transcriptional level. The accumulation of anthocyanins was determined in parallel. The aim of this work was to determine the stage at which the anthocyanin biosynthesis pathway switches off, thus leading to loss of anthocyanins in non-purple carrot cultivars.

## Results

### Taproot color of nine carrot cultivars at different development stages

**Figure 1:**
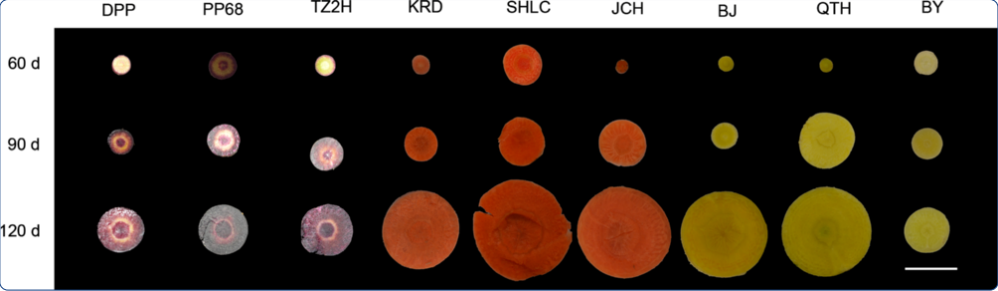
**Colors of the cross-sections of various carrot taproots at three different stages.** Cultivar abbreviations: DPP, Deep purple; PP68, Purple 68; TZ2H, Tianzi2hao; KRD, Kuroda; SHLC, Sanhongliucun; JCH, Junchuanhong; BJ, Bejo1719; QTH, Qitouhuang; BY, Baiyu.

**Figure 2:**
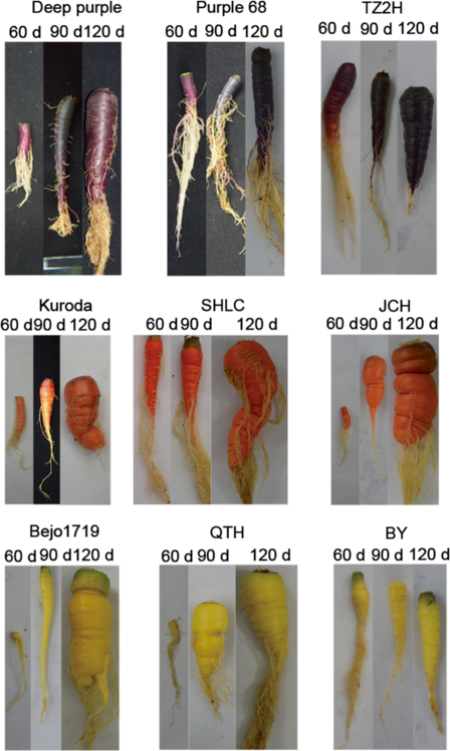
**Epidermis color of the taproots of nine carrots cultivars at three different stages.**

Anthocyanins accumulate in different parts of carrot taproots at different development stages; this leads to taproots displaying a purple or dark color. In 60-day-old carrots, anthocyanins accumulated in the cortex and xylem of ‘Deep purple’ and ‘Purple 68’ cultivar taproots, but only in the cortex of ‘Tianzi2hao’ (Figure 1). In the six other carrot cultivars, no purple or dark coloring was detected in the taproot. In 90- and 120-day-old carrots, anthocyanins accumulated in the cortex, phloem, and xylem of ‘Deep purple’, ‘Purple 68’, and ‘Tianzi2hao’ taproots. In the six other carrot cultivars, no purple or dark coloring was detected in these taproot parts, with the exception of the hypocotyl-derived root part of 90- and 120-day-old ‘Kuroda’, 90-day-old ‘Sanhongliucun’, and 120-day-old ‘Junchuanhong’; these displayed dark color in the epidermis when exposed to light (Figure 2). As hypocotyl-derived parts of the taproots of 120-day-old ‘Sanhongliucun’ and 90-day-old ‘Junchuanhong’ cultivars were not exposed to light, purple or dark coloring was not detectable in their epidermis.

### Anthocyanin content in the taproots of nine carrot cultivars at different developmental stages

**Figure 3:**
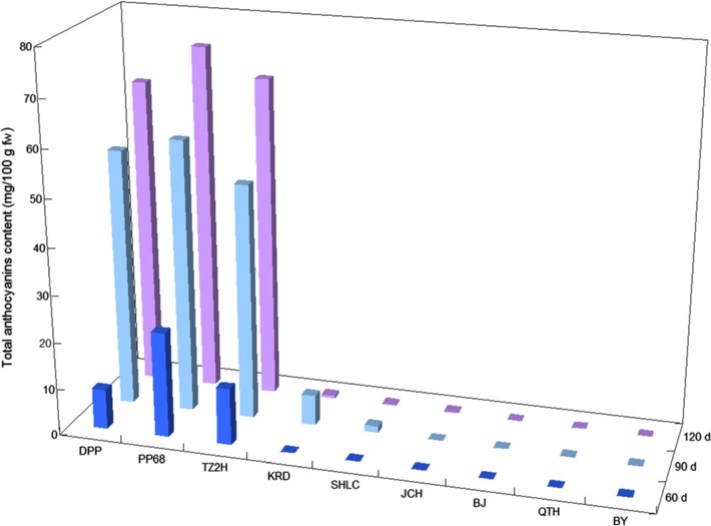
**Total anthocyanin content in the taproot of various carrot cultivars at three different stages.** Values are means of three independent experiments and are calculated as cyanidin 3-*O*-galactoside equivalents.

Total anthocyanin content in the taproots of the three purple carrot cultivars (‘Deep purple’, ‘Purple 68’, and ‘Tianzi2hao’) increased significantly during development (Figure 3). These cultivars accumulated anthocyanins more efficiently in their taproots between the 60- and 90-day-old stages than between the 90- and 120-day-old stages. Of these three purple carrot cultivars, ‘Purple 68’ showed the highest anthocyanin content in the taproots at all three stages. In the taproots of the three orange carrot cultivars (‘Kuroda’, ‘Sanhongliucun’, and ‘Junchuanhong’), anthocyanin was not detected at the 60-day-old stage. At the 90-day-old stage, the anthocyanin content in ‘Kuroda’ and ‘Sanhongliucun’ taproots was 6.47 mg/100 g fresh weight (fw) and 1.35 mg/100 g fw, respectively; anthocyanin was not detected in ‘Junchuanhong’ taproots at this stage. The taproots of 120-day-old ‘Kuroda’, ‘Sanhongliucun’, and ‘Junchuanhong’ contained 0.56, 0.21, and 0.23 mg/100 g fw anthocyanins, respectively. Anthocyanins did not accumulate in the taproots of the three yellow carrot cultivars in any of the three stages.

### Analysis of carrot structural genes for cyanidin-based anthocyanin biosynthesis

**Figure 4:**
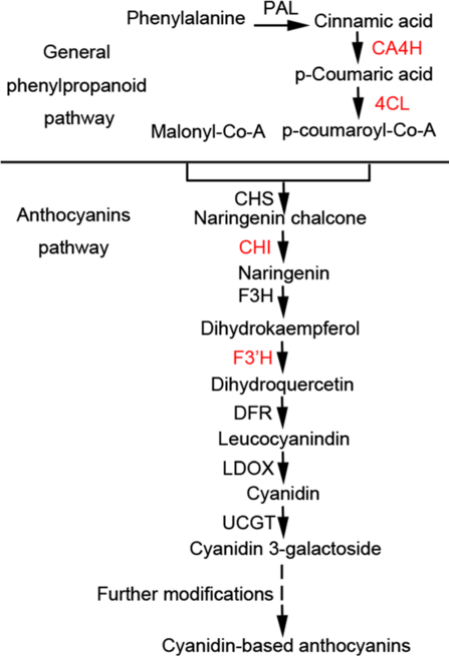
**Schematic of the proposed cyanidin-based anthocyanin biosynthetic pathway.** Enzymes not identified in carrots are marked in red.

As cyanidin-based anthocyanins represent almost all anthocyanin content in carrots, we analyzed structural genes for cyanidin-based anthocyanin biosynthesis. We propose the following cyanidin-based anthocyanin biosynthesis pathway in purple carrots (Figure 4). *PAL*, *CA4H*, and *4CL* code enzymes implicated in the general phenylpropanoid pathway of anthocyanin biosynthesis in carrots. *CHS*, *CHI*, *F3H*, *F3′H*, *DFR*, *LDOX*, and *UCGT* code enzymes involved in the anthocyanin pathway of anthocyanin biosynthesis in carrots. The full names of these genes and their corresponding accession numbers in GenBank and CarrotDB are listed in Table 1 [25].

**Table 1:** **Cyanidin-based anthocyanin biosynthetic genes annotation and accession numbers in GenBank or CarrotDB**

**Gene family**	**Annotation**	**GenBank ID**	**Scaffolds ID in CarrotDB**	**Predict genes ID in CarrotDB**
*PAL*	*PAL1*	D85850.1	scaffold_229, 168181	g443,75570
	*PAL3/PAL4*	AB089813.1/ AB435640.1	scaffold_16648, 16649, 169243	g17456, 17457, 76158
*CA4H*	*CA4H1*	KM359961	scaffold_25749	g24733
	*CA4H2*	KM359962	scaffold_45729	g36870
*4CL*	*4CL1*	KM359963	scaffold_1380	g1988
	*4CL2*	KM359966	scaffold_2619	g3566
*CHS*	*CHS1*	AJ006779.1	scaffold_27333, 21408	g25908, 21339
	*CHS2/ CHS9*	D16255.1/ D16256.1	scaffold_139899, 4932	g61298, 6227
*CHI*	*CHI1*	KM359964	scaffold_8876	g10389
*F3H*	*F3H1*	AF184270.1	scaffold_17226	g17950
*F3'H*	*F3'H1*	KM359965	scaffold_36531	g31974
*DFR*	*DFR1*	AF184271.1	scaffold_1955, 1956	g31520, 2745
*LDOX*	*LDOX1/ LDOX2*	AF184273.1/ AF184274.1	scaffold_20348, 20349, 20350	g20467, 78025, 20472

Gene abbreviations: *PAL*, *Phenylalanine ammonia-lyase*; *CA4H*, *Cinnamate 4-hydroxylase*; *4CL*, *4-coumaroyl-coenzyme A ligase*; *CHS*, *Chalcone synthase*; *CHI*, *Chalcone–flavonone isomerase*; *F3H*, *Flavanone 3-hydroxylase*; *F3′H*, *Flavonoid 3′- hydroxylase*; *DFR*, *Dihydroflavonol 4-reductase*; *LDOX*, *Leucoanthocyanidin dioxygenase*.

*PAL1* (GenBank ID:D85850.1), *PAL3* (GenBank ID:AB089813.1), *PAL4* (GenBank ID:AB435640.1), *CHS1* (GenBank ID:AJ006779.1), *CHS2* (GenBank ID:D16255.1), *CHS9* (GenBank ID:D16256.1), *F3H1* (GenBank ID:AF184270.1), *DFR1* (GenBank ID:AF184271.1), *LDOX1* (GenBank ID:AF184273.1), and *LDOX2* genes (GenBank ID:AF184274.1) were present in GenBank. The *CA4H1*, *CA4H2*, *4CL1*, *4CL2*, *CHI1*, and *F3′H1* genes were identified in CarrotDB using a BLAST-based search tool; these genes were further identified by cloning and sequencing genes from the ‘Deep purple’ cultivar. The nucleotide and deduced amino acid sequences of these genes were deposited at the National Center for Biotechnology Information (NCBI). The NCBI accession numbers of *CA4H1*, *CA4H2*, *4CL1*, *4CL2*, *CHI1*, and *F3′H1* are listed in Table 1.

The *PAL3* and *PAL4*, *CHS2* and *CHS9*, and *LDOX1* and *LDOX2* genes were considered as allelic genes because they share very high identity in their nucleotide acid sequences (>95%); furthermore, only one gene corresponding to each pair of genes was found in the CarrotDB. Therefore, only one pair of primers, specific to each pair of genes, was used for quantitative real-time polymerase chain reaction (qRT-PCR).

### Expression of cyanidin-based anthocyanin biosynthetic genes in the taproot at the 60-day-old stage

**Figure 5:**
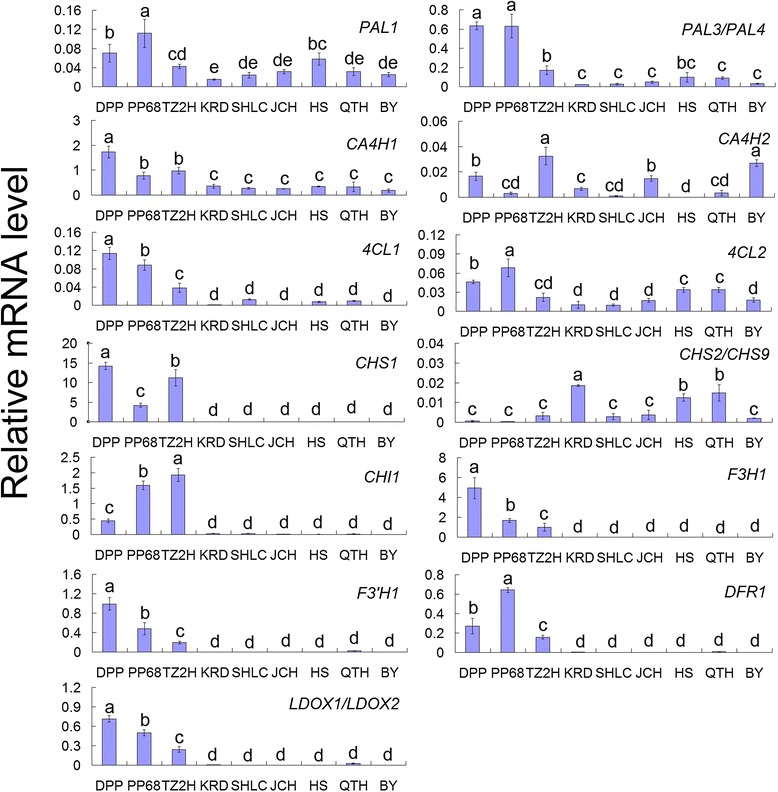
**Expression of cyanidin-based anthocyanin biosynthetic pathway genes in 60-day-old stage carrot taproots.** The mRNA level of *actin1* was defined as 1. Data represents means of biological triplicate qRT-PCRs ± SD. Statistical analysis of differences was performed using Duncan’s multiple range test. Significant differences are indicated by different letters at the *P* < 0.05 level.

Purple carrot cultivars had accumulated anthocyanins in taproots by the 60-day-old stage. Nucleotide sequences of primer pairs used for qRT-PCR specific to each anthocyanin biosynthetic genes are given in Table 2. *PAL3/PAL4*, *CA4H1*, *4CL1*, *CHS1*, *CHI1*, *F3H1*, *F3′H1*, *DFR1*, and *LDOX1/LDOX2* genes showed significantly higher transcript abundance in the taproots of ‘Deep purple’, ‘Purple 68’, and ‘Tianzi2hao’ than in the taproots of ‘Kuroda’, ‘Sanhongliucun’, ‘Junchuanhong’, ‘Bejo1719’, ‘Qitouhuang’, and ‘Baiyu’ (Figure 5). Correlation analysis results revealed that *CHS1*, *CHI1*, *F3H1*, *F3′H1*, *DFR1*, and *LDOX1/LDOX2* were highly correlated with anthocyanin presence among the genes encoding enzymes implicated in the anthocyanin pathway in anthocyanin biosynthesis (Additional file 1: Table S1).

**Table 2:** **Nucleotide sequences of primers specific to cyanidin-based anthocyanin biosynthetic genes and** ***Actin1*** **gene used for qRT-PCR**

**Gene**	**Forward primer 5′-3′**	**Reverse primer 5′-3′**
*PAL1*	ACATTACCCCTTGCTTGCCACT	TCAAAGAATCCACCATCAACTCC
*PAL3/PAL4*	CTGGCATGGCCTCTATGGTACT	TGTCCTGGATGGTGCTTCAACT
*CA4H1*	GTGGAGGCCAACGGAAATG	CGTCCGATTGTGATACCGAGA
*CA4H2*	CCGGCAAAGAGCAAAGTTGT	CAGCCCCGAATGGTAGGAAT
*4CL1*	AAACACCTGCCGTTACACTCG	CGGAAGCAAGATCATCATCGTAT
*4CL2*	AGAGCCAAGTTTCCTAATGCCA	TCCCCGTTGATTCCTTGGTAG
*CHS1*	TTCCACCTTCTCAAAGATGTTCC	GCTCAACTCTGTTTCAACTTGGTC
*CHS2/CHS9*	ATCAGGAAAAGGCAGAGGGC	ATCCGCCTGGTAGACGCAGT
*CHI1*	TCCTGCCACGGTCAAACCT	AAGAGCGAGCGACGGAATC
*F3H1*	GAGTACAGTGAGAAGCTGATGGGTC	GGTTGAGGGCACTTGGGATAG
*F3′H1*	TTGAGGATGGTGAAGGTGGGA	CTTTTGGGCGACGCAGAAC
*DFR1*	GTTATCAAGCCTACCGTACAGGG	AGTTCCAGCAGACGAAGTGTAAAT
*LDOX1/LDOX2*	AGGTGCCCACAGTCGACATAGC	CGCCTGTCCAGCCACTCTAA
*DcActin1*	CGGTATTGTGTTGGACTCTGGTGAT	CAGCAAGGTCAAGACGGAGTATGG

Among the cultivars, ‘Deep purple’ showed the highest taproot mRNA levels of *PAL3/PAL4*, *CA4H1*, *4CL1*, *CHS1*, *F3H1*, *F3′H1*, and *LDOX1/LDOX2*. ‘Purple 68’ had the highest taproot mRNA levels of *PAL1*, *4CL2*, and *DFR1*. ‘Tianzi2hao’ had the highest taproot mRNA levels of *CA4H2* and *CHI1*, and ‘Kuroda’ showed the highest mRNA levels of *CHS2/CHS9* in the taproots.

Transcript levels of *PAL1* and *CA4H2* were lower than *PAL3/PAL4* and *CA4H1*, respectively, in the taproots of all carrot cultivars. Transcript levels of *4CL2* in the taproots of ‘Kuroda’, ‘Junchuanhong’, ‘Bejo1719’, ‘Qitouhuang’, and ‘Baiyu’ were higher than those of *4CL1*, but lower than observed in the taproots of ‘Deep purple’, ‘Purple 68’, ‘Tianzi2hao’, and ‘Sanhongliucun’. In the three purple carrot cultivars, mRNA abundance of *CHS2/CHS9* in the taproot was significantly lower than that of *CHS1*. In the six other carrot cultivars, *CHS2/CHS9* showed similar mRNA expression levels as *CHS1* in the taproots (Figure 5).

## Discussion

In many plants, anthocyanin biosynthesis can be light independent or light induced. The light-independent anthocyanin biosynthesis pathway has been investigated in other plant species, including apple and sweet potato [16,22]. Light-induced anthocyanin biosynthesis has been observed in several plant species, including apple, grape, and Mitchell petunia [18,20,23]. In this work, purple carrot cultivars could produce rich anthocyanins in taproots light independently. In contrast, yellow carrot cultivars failed to produce anthocyanins in the taproots, while orange carrots could only produce small amounts of anthocyanins in the epidermis of hypocotyl-derived root parts light dependently. To determine genes involved in light-independent anthocyanin biosynthesis in purple carrots and those responsible for losses of light-independent anthocyanin production in non-purple carrots, we analyzed anthocyanin pathway structural genes in three purple and six non-purple cultivars.

Some structural genes (*PAL1*, *PAL3/PAL4*, *CHS1*, *CHS2/CHS9*, *F3H1*, *DFR1*, and *LDOX1/LDOX2*) involved in anthocyanin biosynthesis have been previously cloned, and their expression profiles analyzed under ultraviolet light [24,26]. Our previous study identified that *UCGT1* expressed in purple carrot cultivars [25]. In this study, six structural genes (*CA4H1*, *CA4H2*, *4CL1*, *4CL2*, *CHI1*, and *F3′H1*) present in purple (‘Deep purple’) and non-purple (‘Kuroda’) carrot cultivars were cloned and sequenced. *PAL3/PAL4*, *CA4H1*, *4CL1*, *CHS1*, *F3H1*, *F3′H1*, *DFR1*, and *LDOX1/LDOX2* genes may be involved in the anthocyanin biosynthesis in purple carrots as these genes showed higher expression levels in purple carrots than non-purple carrots. *CHS1*, *CHI1*, *F3H1*, *F3′H1*, *DFR1*, and *LDOX1/LDOX2* genes were strongly correlated with the presence of anthocyanins, as indicated by high gene expression levels in the taproots of purple carrot cultivars but no or scarce expression in the taproots of non-purple carrot cultivars. This suggests that these genes predominantly lead to loss of anthocyanin production in non-purple carrot cultivars. A similar result was observed in sweet potatoes [22]. Loss of anthocyanins in the taproots of non-purple carrots is possibly caused by inactivation of regulator genes such as *MYB*, *bHLH*, and *WD40* genes. Future investigation will focus on transcription factors controlling expression of these structural genes to identify the key gene(s) involved in anthocyanin production in purple carrot cultivars and responsible for anthocyanin loss in non-purple carrot cultivars.

The taproots of purple carrots are rich in anthocyanins, reaching a maximum of 175 mg/100 g fw in some cultivars [27]. In this study, anthocyanin content varied significantly in the taproots of the three purple carrot cultivars at the three different stages, as visually indicated by the degree of root coloring. Anthocyanin content in 60-day-old stage taproots of the three purple carrot cultivars was comparable to previously reported for several genotypes of purple carrots [13]. In 90- and 120-day-old purple carrots, anthocyanin content was higher than previously reported, this may be because of the different growth conditions and harvest time of the carrots [13]; anthocyanin accumulation in carrots is sensitive to variations in growth conditions, such as temperature, light, and nutrients [28-30]. Anthocyanin contents in taproots of the three purple carrot cultivars at the 120-day-old stage were higher than those in strawberries, red onion, and red grapes but lower than that observed in blueberries [31]. Anthocyanins accumulated in the epidermis of the hypocotyl-derived root part of the three orange carrot cultivars after they were exposed to light. This suggested that a light-induced anthocyanin biosynthesis pathway is found in these orange carrot cultivars.

## Conclusions

Purple carrot cultivars produced rich amounts of anthocyanins in the taproots light independently, whereas non-purple cultivars did not. The anthocyanin content in purple carrot cultivars increased as root growth occurred. Six novel candidate structural genes that existed in both purple and non-purple carrots were successfully cloned and sequenced. *PAL3/PAL4*, *CA4H1*, *4CL1*, *CHS1*, *CHI1*, *F3H1*, *F3′H1*, *DFR1*, and *LDOX1/LDOX2* may participate in anthocyanin biosynthesis in the taproots of purple carrot cultivars. *CHS1*, *CHI1*, *F3H1*, *F3′H1*, *DFR1*, and *LDOX1/LDOX2* were unexpressed or scarcely expressed in non-purple carrots, thus may lead to the loss of light-independent anthocyanins production in orange and yellow carrots. Our results provide new insights into anthocyanin biosynthesis in carrots at the molecular level and are of importance for other root vegetables.

## Methods

### Plant materials and growth conditions

Three purple carrot cultivars (‘Deep purple’, ‘Purple 68’, and ‘Tianzi2hao’), three orange carrot cultivars (‘Kuroda’, ‘Sanhongliucun’, and ‘Junchuanhong’), and three yellow carrot cultivars (‘Bejo1719’, ‘Qitouhuang’, and ‘Baiyu’) were chosen for this work (Figure 2). Seeds were sown in pots containing a soil/vermiculite mixture (1:1) in a controlled artificial climatic chamber, with a photoperiod of 12 h light (2000–3000 lux) and 12 h dark at day/night temperatures of 22°C/18°C. Carrot plants were grown under the same conditions. Taproots of carrots at 60-, 90-, and 120-day-old stages were harvested, immediately frozen in liquid nitrogen, and stored at −70°C for future analysis. Three taproots were sampled for each carrot cultivar at each stage.

### Determination of anthocyanin content

Carrot taproots were ground to a fine powder in the presence of liquid nitrogen before anthocyanins were extracted. The total anthocyanin content of carrot taproots was determined in accordance with a previously described method [20]. Total anthocyanin quantities were reported in mg cyanidin 3-*O*-galactoside equivalents per 100 g fw (mg/100 g fw). Values were means of three independent experiments.

### RNA isolation and cDNA synthesis

Total RNA was extracted from the taproots of carrots using an RNA Simple Total RNA Kit (Tiangen, Beijing, China) according to manufacturer’s instructions. First-strand cDNA was synthesized from 1 μg of total RNA using a PrimeScript™ RT reagent Kit with a gDNA Eraser (Perfect Real Time) kit (Takara, Dalian, China) following the manufacturer’s protocol. cDNA was diluted 20-fold for gene cloning and qRT-PCR analysis.

### Gene identification, cloning, and sequencing

Nucleotide sequences of genes were searched from the GenBank of the NCBI. Genes not accessed from the NCBI were searched using the genome and transcriptome databases of carrots [25]. Genes were further identified by cloning and sequencing from ‘Deep purple’ in accordance with a previously described method [32].

### qRT-PCR analysis

Primer pairs for qRT-PCR were designed using Primer 5 with a temperature of 59–62°C, length of 19–24 bp, and GC content of 45–55% (Table 2). qRT-PCR was performed on a MyiQ Real-Time PCR Detection System (Bio-Rad) with a SYBR Premix *Ex-Taq* (Takara) in accordance with the manufacturer’s protocol. A total of 20 μL of each reaction contained 10 μL of SYBR Premix *Ex-Taq*, 2 μL of diluted cDNA, 0.2 μM of each primer, and 7.2 μL of ddH_2_O. The qRT-PCR conditions were as follows: denaturation at 95°C for 30 s; 40 cycles of 95°C for 10 s; and 60°C for 30 s. To confirm amplicon purity, melt-curve analysis was performed over a temperature range of 60–95°C at the end of the qRT-PCR. The *DcActin1* gene was used as an internal standard. Experiments were conducted in biological triplicate using three biological RNA samples for each carrot cultivar.

### Statistical analysis

Differences in structural gene expression levels between different carrot genotypes were statistically analyzed using Duncan’s multiple-range test at a 0.05 significance level. Correlation analysis was performed to determine relationships between expression levels of genes encoding enzymes implicated in the anthocyanin pathway of anthocyanin biosynthesis (*CHS1*, *CHS2/CHS9*, *CHI1*, *F3H1*, *F3′H1*, *DFR1*, and *LDOX1/LDOX2*) and anthocyanin presence by logistic regression analysis at a 0.05 significance level.

## Availability of supporting data

The data supporting the results of this article are included within the article.

## Additional file

Additional file 1: Table S1. Correlation between the expression levels of *CHS1*, *CHS2/CHS9*, *CHI1*, *F3H1*, *F3′H1*, *DFR1*, and *LDOX1/LDOX2* and anthocyanin presence by logistic regression analysis.

## Abbreviations

PAL: Phenylalanine ammonia-lyase
CA4H: Cinnamate 4-hydroxylase
4CL: 4-coumaroyl-coenzyme A ligase
CHS: Chalcone synthase
CHI: Chalcone–flavonone isomerase
F3H: Flavanone 3-hydroxylase
F3′H: Flavonoid 3′-hydroxylase
DFR: Dihydroflavonol 4-reductase
LDOX: Leucoanthocyanidin dioxygenase
UCGT: UDP-galactose:cyanidin 3-*O*-galactosyltransferase

## References

[1] He J, Giusti MM (2010). Anthocyanins: natural colorants with health-promoting properties. Annu Rev Food Sci Technol.

[2] Kähkönen MP, Heinonen M (2003). Antioxidant activity of anthocyanins and their aglycons. J Agric Food Chem.

[3] Gould KS (2004). Nature’s Swiss army knife: the diverse protective roles of anthocyanins in leaves. BioMed Res Int.

[4] Shirley BW (1998). Flavonoids in seeds and grains: physiological function, agronomic importance and the genetics of biosynthesis. Seed Sci Res.

[5] Bąkowska-Barczak A (2005). Acylated anthocyanins as stable, natural food colorants–a review. Int J Food Sci Nutr.

[6] Broncel M, Koziróg-Kołacińska M, Andryskowski G, Duchnowicz P, Koter-Michalak M, Owczarczyk A, Chojnowska-Jezierska J (2007). Effect of anthocyanins from *Aronia melanocarpa* on blood pressure, concentration of endothelin-1 and lipids in patients with metabolic syndrome. Pol Merkur Lekarsk.

[7] Hassellund S, Flaa A, Kjeldsen S, Seljeflot I, Karlsen A, Erlund I, Rostrup M (2012). Effects of anthocyanins on cardiovascular risk factors and inflammation in pre-hypertensive men: a double-blind randomized placebo-controlled crossover study. J Hum Hypertens.

[8] Giampieri F, Alvarez-Suarez JM, Tulipani S, Gonzàles-Paramàs AM, Santos-Buelga C, Bompadre S, Quiles JL, Mezzetti B, Battino M (2012). Photoprotective potential of strawberry (*Fragaria* × *ananassa*) extract against UV-A irradiation damage on human fibroblasts. J Agric Food Chem.

[9] Simon PW, Freeman RE, Vieira JV, Boiteux LS, Briard M, Nothnagel T, Michalik B, Kwon Y-S (2008). Carrot. Vegetables II.

[10] Kammerer D, Carle R, Schieber A (2003). Detection of peonidin and pelargonidin glycosides in black carrots (*Daucus carota* ssp. *sativus* var. *atrorubens* Alef.) by high-performance liquid chromatography/electrospray ionization mass spectrometry. Rapid Commun Mass Spectrom.

[11] Turker N, Aksay S, Ekiz Hİ (2004). Effect of storage temperature on the stability of anthocyanins of a fermented black carrot *(Daucus carota* var. L.) beverage: Shalgam. J Agric Food Chem.

[12] Cevallos-Casals BA, Cisneros-Zevallos L (2004). Stability of anthocyanin-based aqueous extracts of Andean purple corn and red-fleshed sweet potato compared to synthetic and natural colorants. Food Chem.

[13] Montilla EC, Arzaba MR, Hillebrand S, Winterhalter P (2011). Anthocyanin composition of black carrot (*Daucus carota* ssp. *sativus* var. *atrorubens* Alef.) cultivars Antonina, Beta Sweet, Deep Purple, and Purple Haze. J Agric Food Chem.

[14] Jaakola L, Määttä K, Pirttilä AM, Törrönen R, Kärenlampi S, Hohtola A (2002). Expression of genes involved in anthocyanin biosynthesis in relation to anthocyanin, proanthocyanidin, and flavonol levels during bilberry fruit development. Plant Physiol.

[15] Kobayashi S, Goto-Yamamoto N, Hirochika H (2004). Retrotransposon-induced mutations in grape skin color. Science.

[16] Espley RV, Hellens RP, Putterill J, Stevenson DE, Kutty-Amma S, Allan AC (2007). Red colouration in apple fruit is due to the activity of the MYB transcription factor, MdMYB10. Plant J.

[17] Matsui K, Umemura Y, Ohme-Takagi M (2008). AtMYBL2, a protein with a single MYB domain, acts as a negative regulator of anthocyanin biosynthesis in Arabidopsis. Plant J.

[18] Albert NW, Lewis DH, Zhang H, Irving LJ, Jameson PE, Davies KM (2009). Light-induced vegetative anthocyanin pigmentation in Petunia. J Exp Bot.

[19] Albert NW, Lewis DH, Zhang H, Schwinn KE, Jameson PE, Davies KM (2011). Members of an R2R3-MYB transcription factor family in Petunia are developmentally and environmentally regulated to control complex floral and vegetative pigmentation patterning. Plant J.

[20] Li Y-Y, Mao K, Zhao C, Zhao X-Y, Zhang H-L, Shu H-R, Hao Y-J (2012). MdCOP1 ubiquitin E3 ligases interact with MdMYB1 to regulate light-induced anthocyanin biosynthesis and red fruit coloration in apple. Plant Physiol.

[21] Schaart JG, Dubos C, Romero De La Fuente I, Houwelingen AM, Vos RC, Jonker HH, Xu W, Routaboul JM, Lepiniec L, Bovy AG (2013). Identification and characterization of MYB-bHLH-WD40 regulatory complexes controlling proanthocyanidin biosynthesis in strawberry (*Fragaria* × *ananassa*) fruits. New Phytol.

[22] Mano H, Ogasawara F, Sato K, Higo H, Minobe Y (2007). Isolation of a regulatory gene of anthocyanin biosynthesis in tuberous roots of purple-fleshed sweet potato. Plant Physiol.

[23] Takos AM, Jaffé FW, Jacob SR, Bogs J, Robinson SP, Walker AR (2006). Light-induced expression of a MYB gene regulates anthocyanin biosynthesis in red apples. Plant Physiol.

[24] Hirner AA, Veit S, Seitz HU (2001). Regulation of anthocyanin biosynthesis in UV-A-irradiated cell cultures of carrot and in organs of intact carrot plants. Plant Sci.

[25] Xu Z-S, Tan H-W, Wang F, Hou X-L, Xiong A-S (2014). CarrotDB: a Genomic and Transcriptomic Database for Carrot. Database.

[26] Glässgen WE, Rose A, Madlung J, Koch W, Gleitz J, Seitz HU (1998). Regulation of enzymes involved in anthocyanin biosynthesis in carrot cell cultures in response to treatment with ultraviolet light and fungal elicitors. Planta.

[27] Mazza G, Miniati E (1993). Anthocyanins in Fruits, Vegetables, and Grains.

[28] Dougall D, LaBrake S, Whitten G (1983). The effects of limiting nutrients, dilution rate, culture pH, and temperature on the yield constant and anthocyanin accumulation of carrot cells grown in semicontinuous chemostat cultures. Biotechnol Bioeng.

[29] Takeda J, Abe S (1992). Light-induced synthesis of anthocyanin in carrot cells in suspension—IV. the action spectrum. Photochem Photobiol.

[30] Kırca A, Özkan M, Cemeroğlu B (2007). Effects of temperature, solid content and pH on the stability of black carrot anthocyanins. Food Chem.

[31] Wu X, Beecher GR, Holden JM, Haytowitz DB, Gebhardt SE, Prior RL (2006). Concentrations of anthocyanins in common foods in the United States and estimation of normal consumption. J Agric Food Chem.

[32] Jiang Q, Xu Z-S, Wang F, Li M-Y, Ma J, Xiong A-S (2014). Effects of abiotic stresses on the expression of *Lhcb1* gene and photosynthesis of *Oenanthe javanica* and *Apium graveolens*. Biol Plantarum.

