# Supplementary material for: Transcript profiling of structural genes involved in cyanidin-based anthocyanin biosynthesis between purple and non-purple carrot (Daucus carota L.) cultivars reveals distinct patterns
Source: BMC Plant Biol. 2014 Oct 1;14:262. doi: 10.1186/s12870-014-0262-y (PMC4190390; doi:10.1186/s12870-014-0262-y)
Supplement: Additional file 1: Table S1. — Correlation between the expression levels of CHS1, CHS2/CHS9, CHI1, F3H1, F3′H1, DFR1, and LDOX1/LDOX2 and anthocyanin presence by logistic regression analysis. [file 12870_2014_262_MOESM1_ESM.doc]

**Table S1**

Correlation between the expression levels of *CHS1*, *CHS2/CHS9*, *CHI1*, *F3H1*, *F3’H1*, *DFR1*, and *LDOX1/LDOX2* and anthocyanin production using logistic regression analysis.

| Gene | Score | Significance |
| --- | --- | --- |
| *CHS1* | 7.084 | 0.008 |
| *CHS2/CHS9* | 2.815 | 0.093 |
| *CHI1* | 6.652 | 0.010 |
| *F3H1* | 5.343 | 0.021 |
| *F3’H1* | 5.864 | 0.015 |
| *DFR1* | 5.950 | 0.015 |
| *LDOX1/LDOX2* | 7.229 | 0.007 |
